# The Impact of Montelukast Duration on the Risk of Neuropsychiatric Disorders in Children with Asthma: A Population-Based Cohort Study

**DOI:** 10.3390/ph18030379

**Published:** 2025-03-07

**Authors:** Wei-Te Lei, Chien-Yu Lin, Szu-Hung Chu, Li-Ching Fang, Yu-Hsuan Kao, Po-Li Tsai, Yu-Wen Lin, Fung-Chang Sung, Shu-I Wu

**Affiliations:** 1 Division of Immunology, Rheumatology, and Allergy, Department of Pediatrics, Hsinchu Municipal MacKay Children’s Hospital, Hsinchu 30070, Taiwan; weite.lei@gmail.com (W.-T.L.); 4525@mmh.org.tw (S.-H.C.); 2 Division of Infectious Disease, Department of Pediatrics, Hsinchu Municipal MacKay Children’s Hospital, Hsinchu 30070, Taiwan; mmhped.lin@gmail.com; 3 Division of Immunology, Rheumatology, and Allergy, Department of Pediatrics, MacKay Children’s Hospital, Taipei 10449, Taiwan; fang04@gmail.com (L.-C.F.); evakao65@gmail.com (Y.-H.K.); 4 Division of Colorectal Surgery, Department of Surgery, MacKey Memorial Hospital, Taipei 10449, Taiwan; tpoli0503@gmail.com; 5 Department of Medical Research, MacKay Memorial Hospital, Taipei 10449, Taiwan; yuwen1014@gmail.com; 6 Graduate Institute of Clinical Medical Science, China Medical University, Taichung 404333, Taiwan; fcsung@mail.cmu.edu.tw; 7 Management Office for Health Data, China Medical University Hospital, Taichung 40447, Taiwan; 8 Department of Health Services Administration, China Medical University, Taichung 404333, Taiwan; 9 Department of Medicine, MacKay Medical College, New Taipei City 25200, Taiwan; 10 Section of Psychiatry and Suicide Prevention Center, MacKay Memorial Hospital, Taipei 10449, Taiwan

**Keywords:** montelukast, neuropsychiatric disorders, pediatric asthma, Tics/Tourette’s syndrome

## Abstract

**Background/Objectives:** Asthma is one of the most common chronic diseases in children, and montelukast is widely prescribed to manage symptoms. However, concerns have emerged regarding its potential association with neuropsychiatric disorders. This study aims to investigate the impact of montelukast duration on neuropsychiatric risks in children with asthma. **Methods:** A cohort study was conducted using Taiwan’s National Health Insurance Research Database (NHIRD), including children diagnosed with asthma between 2004 and 2007. A total of 14,606 children in the montelukast cohort and 8432 in the non-montelukast cohort were analyzed, with propensity score matching applied to reduce confounding bias. Neuropsychiatric outcomes, including Tics/Tourette’s syndrome, were evaluated using Cox proportional hazard models. **Results:** Overall, montelukast use did not increase the risk of neuropsychiatric disorders. However, among children aged 6–15 years, prolonged use beyond 63 days was associated with a significantly elevated risk of Tics/Tourette’s syndrome, with a 2.6-fold increase observed in girls and a 1.8-fold increase in boys. Conversely, shorter montelukast use in children aged 0–6 years was linked to a lower risk of neuropsychiatric disorders. **Conclusions:** Although montelukast generally does not elevate neuropsychiatric risks, extended use in older children may increase the likelihood of developing Tics/Tourette’s syndrome. These findings highlight the importance of cautious prescribing in pediatric asthma management. Further research is necessary to validate these associations and inform clinical decision making.

## 1. Introduction

Asthma is one of the most common chronic diseases in children, characterized by reversible airflow obstruction, bronchial hyperresponsiveness, and chronic inflammation. The global prevalence of asthma is approximately 5.8% [1]. The disease can lead to missed school days, reduced quality of life, hospitalization, and even death in severe cases. Overall, the disability-adjusted life years (DALYs) for childhood asthma in different age groups ranged from 0.95 to 1.49 per 1,000,000 [2].

Currently, there are various medications available for the treatment of asthma. Leukotriene-modifying agents (LTMAs), by blocking the cysteinyl leukotriene receptor, can prevent the inflammatory response in the airways caused by leukotrienes and their derivatives [3]. According to the current GINA guidelines, while LTRA is not recommended as a first-line treatment for adolescents aged 12 years and older and adults, it remains an alternative controller option at STEP 2 and STEP 3 for children aged 5 years and younger and 6–11 years, as well as an alternative add-on therapy at STEP 4 [4]. Montelukast is the only leukotriene receptor antagonist (LTRA) approved for use in children aged 6 months and older, as well as those under 12 years old. Previous studies have demonstrated that montelukast can reduce the risk of asthma exacerbations in children with mild intermittent asthma and improve lung function [5,6,7,8]. However, some reports have indicated that montelukast may be associated with psychiatric side effects [9].

Since March 2008, there have been concerns about a possible risk of neuropsychiatric disease, including sleep disorders, hyperactivity, and anxiety, during montelukast use in children [10]. In 2009, the product label included warnings for agitation, aggressive behavior or hostility, anxiousness, depression, disorientation, disturbance in attention, dream abnormalities, hallucinations, insomnia, irritability, memory impairment, restlessness, somnambulism, suicidal thinking and behavior, and tremor, and has been formerly called a black box warning by the FDA [11]. Mechanistically, in addition to being a well-known CysLTR1 antagonist, montelukast is also an inhibitor of 5-lipoxygenase (5-LOX) and an antagonist of CysLTR2, P2Y12, and GPR17 receptors. These receptors have been implicated in various pathological mechanisms and may hold therapeutic potential, particularly in central nervous system disorders such as Parkinson’s disease and Alzheimer’s disease [12,13,14,15]. Thus, montelukast may exert both therapeutic and neurotoxic effects. Previous multi-omics studies have indicated that montelukast can influence neurotransmitter pathways, especially by modulating the hypothalamic–pituitary–adrenal (HPA) axis [16]. This provides pathophysiological evidence supporting montelukast’s potential role in neuropsychiatric disorders. However, there is conflicting evidence regarding the association between montelukast and neuropsychiatric disease [9,10,17,18,19,20,21,22,23,24,25,26]. Although analyses of clinical data suggest no strong association between montelukast and neuropsychiatric disorders, some evidence has indicated a potential link, particularly in children and adolescents. Furthermore, in the pediatric population, newly diagnosed neuropsychiatric disorders are often attributed to new or ongoing medication use [27,28]. However, it is difficult to establish a causal relationship, and previous observational studies have mostly been case reports, lacking strong empirical evidence. To further explore this possible association, we conducted a study using a nationwide research database to examine the relationship between montelukast and neuropsychiatric effects in children with asthma in Taiwan.

## 2. Results

### 2.1. Baseline Characteristics in Montelukast and Non-Montelukast Cohorts

**Figure 1:**
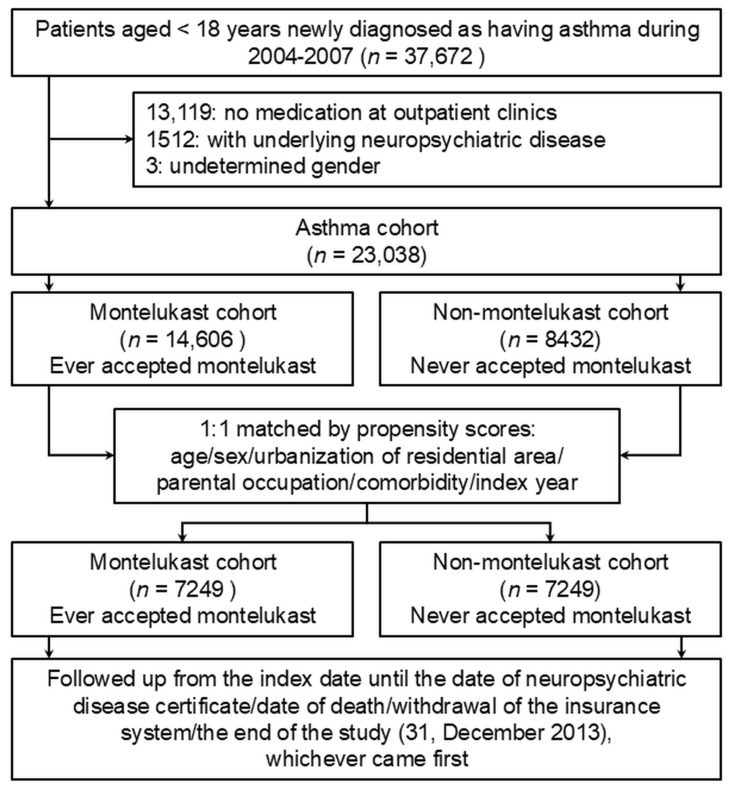
Diagram of patient study flow.

A total of 37,672 children newly diagnosed with asthma were identified from the database between 1 January 2004 and 31 December 2007. After applying rigorous criteria, we selected 14,606 patients for the montelukast cohort and 8432 patients for the non-montelukast cohort. Following 1:1 matching by propensity scores, which included factors such as age, sex, urbanization of residential area, parental occupation, and comorbidities, 7249 patients remained in each cohort (Figure 1). The basal differences before the propensity score matching can be seen in Table S1.

Table 1 lists the demographic characteristics of the patients in each group. The percentage of males and the mean age are similar between the two groups, at approximately 59% and 6.55 years, respectively. The distribution of patients across three different age groups also shows no significant statistical difference between the cohorts. To specifically assess the potential contribution of montelukast to the development of psychiatric disorders, we meticulously removed possible confounding factors, including both allergic and non-allergic comorbidities, as well as other chronic diseases that could influence mood and mental health. These factors included allergic rhinitis, atopic dermatitis, convulsions, hypothyroidism, epilepsy, meningitis, juvenile idiopathic arthritis, dermatomyositis, ankylosing spondylitis, Kawasaki disease, and child abuse. After thorough matching, the only significant difference between the two groups was a higher percentage of inhaled corticosteroid (ICS) and ICS/long-acting beta-2 agonist (LABA) use in the non-montelukast group.

### 2.2. Influence of Montelukast on the Incidence of Psychiatric Disease

**Figure 2:**
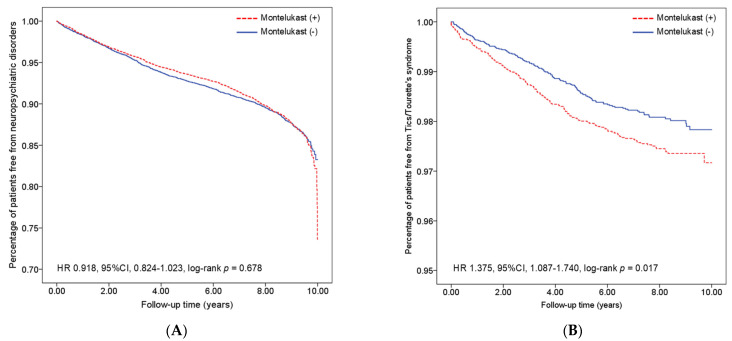
Kaplan–Meier analysis of the two groups. The analysis showed (**A**) no significant difference in neuropsychiatric disease-free rates between groups (log-rank test, *p* = 0.678) and (**B**) a significantly lower disease-free rate for Tics/Tourette’s syndrome (log-rank test, *p* = 0.017) in montelukast users.

Table 2 lists the incidence rates and hazard ratios of various psychiatric disorders based on univariate Cox proportional analysis between the two cohorts. Overall, there was no statistically significant difference in the incidence rates of the listed neuropsychiatric disorders between the two groups (Figure 2A, 89.1% in the exposed group versus 89% in the non-exposed group, respectively), except for Tics/Tourette’s syndrome (Figure 2B). The hazard ratio for Tics/Tourette’s syndrome in the montelukast cohort was 1.375 (95% CI 1.087–1.740, *p* = 0.008) compared to the non-montelukast cohort.

### 2.3. Influence of Age, Sex, Asthma Control Status, and Duration of Montelukast Use on Neuropsychiatric Disease in Children with Asthma

**Figure 3:**
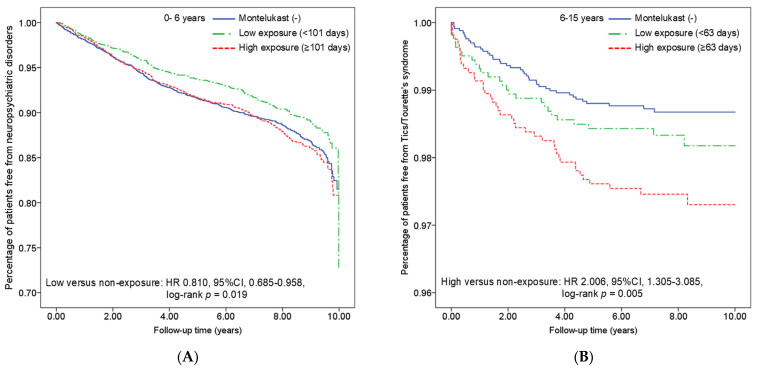
The Kaplan–Meier analysis showed (**A**) children aged 0–6 years with less than 101 days of montelukast had a significantly lower neuropsychiatric disorder-free rate (*p* = 0.019) and (**B**) children aged 6–15 years with more than 63 days of montelukast had a lower Tics/Tourette’s syndrome-free rate (*p* = 0.005).

To further delineate the population most likely affected by montelukast use, we stratified the data by age, corresponding to the different dosages recommended for montelukast. The age groups were divided as follows: under 6 years, 6 to 15 years, and 15 to 18 years. Additionally, we categorized exposure duration into high-dose and low-dose groups based on the median number of days of montelukast use within each age category. The median days of use for each age group were 101 days, 63 days, and 56 days, respectively (Table S2). Previous studies have shown that the degree of asthma control status and the use of corticosteroids may both contribute to the development of Tics/Tourette’s syndrome and other neuropsychiatric disorders [29,30,31]. Therefore, we further adjusted for asthma control status in our analysis. Table S3 includes the asthma control status in patients who were classified as well controlled and not well controlled, with and without the use of montelukast. Table 3 presents the results of the impact of different montelukast exposure durations on subsequent neuropsychiatric disorders across different age groups. In the 6–15 and 15–18 age groups, the conclusion remains unchanged: the use of montelukast does not increase the risk of subsequent neuropsychiatric disorders. Interestingly, in children under 6 years old, those with fewer days of montelukast use (<101 days) showed a lower risk of developing neuropsychiatric disorders later, with an adjusted hazard ratio of 0.813 (95% CI 0.688–0.961, *p* < 0.05). Figure 3A showed the disease-free rate at the end of the follow-up period (89.7% for the low exposure group versus 87.3% in the high exposure group, *p* = 0.019). This effect is even more pronounced in boys aged 0–6 years, with an adjusted hazard ratio of 0.726 (95% CI 0.594–0.888, *p* < 0.01) (Table S4). However, in girls aged 0–6 years, there was no statistically significant difference observed (Table S4).

### 2.4. The Effect of Montelukast on Subsequent Tics/Tourette’s Syndrome in Children with Asthma

We then focused on the impact of montelukast use on the subsequent development of Tics/Tourette’s syndrome across different age groups and genders. We found that in asthmatic children aged 6–15 years, using montelukast for more than 63 days increases the risk of developing Tics/Tourette’s syndrome later, regardless of how well their asthma is controlled. Figure 3B shows the disease-free rate in each group at the end of the 10-year follow-up. The crude hazard ratio was 2.006 (95% CI 1.305–3.085, *p* < 0.01), and the adjusted hazard ratio was 1.998 (95% CI 1.299–3.075, *p* < 0.01) (Table 4). Intriguingly, when further stratified by gender and adjusted for asthma control status, this effect remained significant in both boys and girls, with a higher risk observed in females: the adjusted hazard ratio was 1.842 (95% CI 1.123–3.022, *p* < 0.05) for boys and 2.567 (95% CI 1.063–6.199, *p* < 0.01) for girls (Table S5). To confirm that the occurrence of Tics/Tourette’s syndrome was not influenced by corticosteroid dosage or duration, we further analyzed these factors within the montelukast cohort. The results showed no significant differences between those who developed Tics/Tourette’s syndrome and those who did not (Table S6).

## 3. Discussion

To the best of our knowledge, this is the first population-based cohort study to comprehensively investigate the impact of montelukast dosage on the risk of subsequent neuropsychiatric diseases in children with asthma. This study demonstrated that, overall, the use of montelukast does not increase the risk of developing neuropsychiatric disorders in children, except for Tics/Tourette’s syndrome. Furthermore, regardless of asthma control, asthmatic boys aged 0 to 6 years who used montelukast for fewer than 101 days had approximately a 28% reduced risk of developing neuropsychiatric diseases compared to those who did not use it. Conversely, in children aged 6 to 15 years, using montelukast for more than 63 days increased the risk of developing Tics/Tourette’s syndrome compared to non-users, regardless of gender, with girls having a 2.6-fold higher risk and boys a 1.8-fold higher risk.

Montelukast is the most potent leukotriene receptor antagonist (LTRA) and achieves its therapeutic effect in asthma by selectively blocking the cysteinyl leukotriene receptor type 1 (CysLT1R) on the smooth muscle of the lungs [32,33]. CysLT1R is also widely expressed in other cells throughout the body, such as macrophages in the lungs, monocytes in peripheral blood, and brain tissue. Studies on positive effects of montelukast in a preterm mouse model of encephalopathy of prematurity and in adult cognitive function have also demonstrated its ability to cross the blood–brain barrier [34,35]. Nevertheless, an in vitro study proved that there is a direct toxic effect of montelukast on microglial and neuronal cells caused by an inflammatory response by prostaglandin E2 (PGE2) and reactive oxygen species (ROS), suggesting the possible pathophysiologic cause of neuropsychiatric events in the users [36].

Chronic diseases can impact a patient’s mental health, and this effect is less frequently studied in children, particularly in younger age groups where such diagnoses are rarer. Previous research has indicated that children aged 6 to 16 years with chronic illnesses persisting for more than three years are at an increased risk of developing neuropsychiatric comorbidities [37]. Research on children with asthma has also found that those with severe persistent asthma and poorly controlled asthma have a significantly higher prevalence of oppositional defiant disorder (ODD), separation anxiety disorder (SAD), and ADHD, as well as a greater likelihood of experiencing their first depressive episode in childhood [38,39]. In the current study, it was observed that in the 0–6 age group, children who used montelukast for a shorter duration had a lower risk of developing neuropsychiatric disorders later, and this result remained consistent even after adjusting for asthma severity. One possible explanation is that a shorter duration of use reflects a shorter course of asthma, which aligns with observations from previous research, rather than indicating that less montelukast use directly reduces the risk of future neuropsychiatric disorders. Overall, this study adds evidence that using montelukast in this age group does not increase the risk of subsequent neuropsychiatric disorders.

Tics/Tourette’s syndrome is a group of childhood-onset neurodevelopmental disorders characterized by multiple motor or vocal tics. These disorders typically begin before the age of 5, peak in severity between 8 and 12 years, and gradually improve into adulthood [40]. The exact mechanisms underlying Tics/Tourette’s syndrome are currently unknown, but the disorder is likely associated with genetic, environmental, and immunological factors [41]. The positive association with allergic diseases has been extensively studied [42]. The possible immune and neuroimmunomodulatory mechanisms underlying the correlation between Tics/Tourette’s syndrome and allergic diseases are varied. They include increased levels of interleukin-12 and tumor necrosis factor-alpha, immune dysregulation caused by infectious diseases and inflammatory responses, and mucosal hyperresponsiveness due to the interaction of immune cells with neuropeptides and neurotransmitters released by the nervous system [43,44,45,46]. Although montelukast did not lead to an overall increase in neuropsychiatric diseases, the current study found that in the 6–15 age group, longer use (more than 63 days) was associated with an increased risk of developing Tics/Tourette’s syndrome later. Although ICS/LABA and OCS may themselves cause emotional issues, including effects on emotion, mood, and sleep [47], and have been linked to Tics/Tourette’s syndrome in a few case reports [48], the population using montelukast in this study was already less likely to use ICS or ICS/LABA at the time of the matching step (Table 1). One might also question whether severe asthma could increase the risk of neuropsychiatric disorders, thereby introducing bias into the study. Montelukast might be used as an add-on therapy for treating severe asthma, which could further complicate the analysis. To address this potential bias, we adjusted for asthma severity in our analysis, and the conclusions remained consistent, reinforcing that the impact of montelukast on this age group is indeed present.

### Strengths and Limitations

A key strength of this study is the use of a nationwide, population-based dataset, which is large enough to provide robust statistical power for detecting the associations of interest. The unique characteristics of the data source enhance the reliability of the estimated disease incidences [49]. However, there were still several limitations in this study. First, although we made every effort to minimize bias by matching factors such as sex, age, urbanization, parental income, and comorbidities, and by adjusting for covariates like asthma severity, we were unable to account for certain unrecorded variables in the NHIRD. These include serum IgE and specific IgE levels, eosinophil counts, infection pathogens, and montelukast-associated genetic factors that might be related to neuropsychiatric symptoms [50]. Second, this population-based study primarily focused on the ethnic group in Taiwan, so the findings may not be fully applicable to other populations. Third, we did not include all neuropsychiatric disorders, such as obsessive–compulsive disorder (OCD), post-traumatic stress disorder (PTSD), and developmental coordination disorder (DCD), which may lead to overlooking montelukast’s effects on certain conditions. However, the neuropsychiatric disorders we included are those most commonly observed in the pediatric population. Fourth, the current study did not track the duration of montelukast use after the diagnosis of neuropsychiatric disorders, whether the medication was discontinued, or the severity, duration, or prognosis of neuropsychiatric diseases following their development after montelukast use. Therefore, we were unable to assess the medium- to long-term impact of montelukast on neuropsychiatric diseases. Lastly, it has been reported that neuropsychiatric disorders have genetic factors and familial predisposition. However, the current study lacks data on the neuropsychiatric disorder history of parents or other family members, as well as relevant genetic testing reports, limiting our ability to further explore this aspect. Therefore, the current study indicates that the observed association between prolonged montelukast use in children aged 6–15 years and an increased risk of developing Tics/Tourette’s syndrome is an epidemiological finding, rather than a mechanistic explanation.

## 4. Materials and Methods

The Taiwan National Health Insurance Research Database (NHIRD) releases a set of sampling files, called the Longitudinal Health Insurance Database (LHID), for research purposes. LHID 2000 contains the original claims data for 1,000,000 beneficiaries randomly sampled from the entire population of NHI beneficiaries in the year 2000. All registration and claims data for these 1,000,000 individuals for the period of 1997–2012 were collected and distributed as LHID 2000. The subjects included in LHID 2000 and those enrolled in the original Taiwan NHIRD do not differ significantly in age, sex, or mean insured amount [51]. A cohort with asthma and non-asthma comparison groups was developed. We identified people with a verified, new diagnosis of asthma in 2000. The definition of asthma diagnosis is to have at least 2 diagnoses at outpatient clinics and to be prescribed at least 1 kind of asthma controller medication (e.g., montelukast, ICS, ICS+LABA). The non-asthma group was made up of those who did not have asthma from 1997 to 2012 and was matched 4:1 with the asthma group by sex, age, and index date. The index date was defined as the date when the patients were first diagnosed as having asthma. Any individuals that had neuropsychiatric disease prior to the index date were excluded from this study. The two study groups were followed up from the index date until the date of issuance of neuropsychiatric disease certificates, the date of death, or the end of the study (31 December 2012), whichever came first. All data were calculated with SPSS 18.0 software for descriptive statistics and contingency tables (SPSS, Inc., Chicago, IL, USA). Kaplan–Meier survival analysis and Cox regression were used. A value of *p* = 0.05 was considered statistically significant.

### 4.1. Database

On 1 March 1995, the National Health Insurance (NHI) was established in Taiwan, achieving a coverage rate exceeding 99% among Taiwanese citizens. Each year, the Bureau of NHI provides data containing encrypted personal identifications, diagnoses, and healthcare utilizations to compose the Taiwan NHIRD [52]. In the NHIRD, diseases are coded according to the International Classifications of Disease, Ninth Revision, Clinical Modification (ICD-9-CM). A subset of the NHIRD, the Longitudinal Health Insurance Database (LHID), was used in our analysis. The LHID contains 3 million randomly selected enrollees from the NHIRD. Distributions of age, gender, and healthcare costs between the LHID and the general population in Taiwan are not significantly different [53]. This study was reviewed and approved by the institutional Review Board of China Medical University, Taichung, Taiwan (IRB approval number: CMUH104-REC2-115(CR-3)). The institutional review board exempted consent requirements.

### 4.2. Study Sample

A cohort with asthma and non-asthma comparison groups was developed. We identified people with a verified, new diagnosis of asthma in 2000. The definition of asthma diagnosis is to have at least 2 diagnoses (International Classification of Diseases, ninth edition, Clinical Modification (ICD-9-CM) codes: 49390) at either emergency departments or outpatient clinics, and individuals hospitalized to be prescribed at least 1 kind of asthma controller medication (e.g., montelukast, ICS, ICS+LABA). The non-asthma group was made up of those who did not have asthma from 1997 to 2012 and were matched 4:1 with the asthma group by sex, age, and index date. The index date was defined as the date when the patients were first diagnosed as having asthma. Any individuals that had neuropsychiatric disease prior to the index date were excluded from the study. The two study groups were followed up from the index date until the date of issuance of neuropsychiatric disease certificates, the date of death, or the end of the study (31 December 2012), whichever came first. The flow chart of the study patients is shown in Figure 1. Neuropsychiatric disease was defined as occurrence of any one of the following diagnoses: ADHD (attention deficit hyperactivity disorder), affective disorder, anxiety disorders, autism, autistic spectrum disorder, conduct disorders, delay, emotional disturbance (childhood and adolescent), personality disorder, psychosis, adjustment disorder, suicide, schizophrenia, and Tics/Tourette’s syndrome. The comorbidity variables include allergic rhinitis, atopic dermatitis, convulsions, epilepsy, congenital hypothyroidism, meningitis, juvenile idiopathic arthritis, systemic lupus erythematous, dermatomyositis, ankylosing spondylosis, Kawasaki disease, and child abuse. The International Classification of Diseases, Ninth edition, Clinical Modification (ICD-9-CM) codes are listed in Table S0. To simultaneously account for the effects of asthma control status and corticosteroid use on neuropsychiatric disorders, asthma control status was defined based on systemic corticosteroid use as follows: Asthma was classified as “not well controlled” if the patient had more than two visits per year to the emergency department with a diagnosis of asthma and received oral or injectable corticosteroids, “or” was hospitalized for asthma with corticosteroid treatment, “or” had outpatient asthma diagnoses with prescriptions for oral corticosteroids. If the patient had 0–1 such events per year, their asthma was considered to be better controlled.

### 4.3. Statistical Analysis

Data analysis was conducted using SPSS 18.0 software for descriptive and contingency tables (SPSS, Inc., Chicago, IL, USA). Pearson’s chi-squared tests were used for comparisons of categorical variables. Kaplan–Meier survival analysis and Cox proportional hazard models were used to estimate hazard ratios and adjust for other covariates. *p* < 0.05 was considered statistically significant.

## 5. Conclusions

This large population-based cohort study comprehensively examined the relationship between montelukast duration and the risk of neuropsychiatric disorders in children with asthma. Our findings indicate that, overall, montelukast use does not increase the risk of neuropsychiatric disorders. However, in children aged 6–15 years, prolonged use of montelukast beyond 63 days was associated with a higher risk of developing Tics/Tourette’s syndrome. Conversely, in children 0–6 years old, shorter montelukast use (<101 days) was linked to a lower risk of developing neuropsychiatric disorders, particularly in boys. These results emphasize the need for cautious prescribing practices, particularly regarding prolonged montelukast use in older children, while also highlighting its potentially safer profile in younger children. Given the observational nature of this study, further research—including prospective studies and genetic investigations—is needed to validate these associations and provide more definitive clinical guidance.

## Figures and Tables

**Table 1:** Demographics and comorbidity differences between patients receiving montelukast integration treatments (montelukast cohort) and those who are not (non-montelukast cohort) in children with asthma after propensity score matching.

	Asthmatic Children				
	Propensity Score Matching				
	Non-Montelukast Cohort		Montelukast Cohort		
	N = 7249		N = 7249		*p*
Variables	n	%	n	%	
Sex					
Girls	2978	41.1	2971	40.0	0.906
Boys	4271	58.9	4278	59.0	
Age, year, mean (SD)	6.55 (±3.45)		6.53 (±3.34)		0.799
<6	3709	51.2	3809	52.4	0.175
≥6, <15	3333	46.0	3255	44.9	
≥15	207	2.9	185	2.6	
Index year					
2004	2001	27.6	2045	28.2	0.934
2005	1889	26.1	1880	25.9	
2006	1383	19.1	1366	18.8	
2007	1164	16.1	1165	16.1	
2008	812	11.2	793	10.9	
Urbanization					
Urban	4300	59.3	4317	59.6	0.487
Rural	2177	30.0	2125	29.3	
Offshore regions	772	10.6	807	11.1	
Parents’ salary					
<15,840	7075	97.6	7109	98.1	0.140
15,841–25,000	134	1.8	105	1.4	
>25,001	40	0.6	35	0.5	
Comorbidity					
Allergic rhinitis	5371	74.1	5358	73.9	0.806
Atopic dermatitis	1397	19.3	1354	18.7	0.362
Convulsion	36	0.5	41	0.6	0.568
Hypothyroidism	13	0.2	11	0.2	0.683
Epilepsy	83	1.1	89	1.2	0.645
Meningitis	39	0.5	40	0.6	0.910
JIA	2	<0.1	2	<0.1	>0.999
SLE	4	0.1	3	<0.1	>0.999
Dermatomyositis	0	N/A	0	N/A	N/A
Spondylitis	0	N/A	0	N/A	N/A
Kawasaki disease	39	0.5	41	0.6	0.823
Abuse	0	N/A	0	N/A	N/A
Asthma medication used					
ICS	7227	99.7	2909	40.1	<0.001
ICS/LABA	1144	15.8	630	8.7	<0.001

SD: standard difference, JIA: juvenile idiopathic arthritis, SLE: systemic lupus erythematosus, ICS: inhaled corticosteroid, LABA: long-acting beta-2 agonist, N/A: not applicable.

**Table 2:** Incidence and hazard ratios of disorders stratified by sex, age, comorbidity, and asthma medication used between non-montelukast and montelukast cohorts, after propensity score matching.

	Asthmatic Children							
	Non-Montelukast Cohort			Montelukast Cohort				
Variables	Event	PY	Rate	Event	PY	Rate	Hazard Ratio (95% CI)	*p*
Overall disorder	799	67,314.214	11.87	788	67,575.178	11.66	0.918 (0.824–1.023)	0.123
ADHD	151	71,431.646	2.11	122	71,612.871	1.70	0.832 (0.653–1.061)	0.139
Adjustment disorder	84	71,975.321	1.17	67	72,105.803	0.93	0.782 (0.559–1.093)	0.150
Affective disorder	34	72,287.028	0.47	48	72,207.289	0.66	1.333 (0.831–2.141)	0.234
Anxiety	322	70,460.28	4.57	302	70,663.252	4.27	0.901 (0.761–1.067)	0.228
Autism	29	72,294.277	0.40	19	72,352.269	0.26	0.708 (0.381–1.318)	0.277
Conduct	0	N/A	N/A	1	N/A	N/A	N/A	N/A
Delay	229	70,713.995	3.24	190	71,040.2	2.67	0.821 (0.674–1.001)	0.051
Emotional disorder	121	71,707.108	1.69	102	71,786.847	1.42	0.825 (0.628–1.083)	0.166
Personality disorder	3	72,461.004	0.04	3	72,461.004	0.04	1.000 (0.202–4.955)	>0.99
Psychosis	3	72,461.004	0.04	6	72,453.755	0.08	2.000 (0.500–7.997)	0.327
Schizophrenia	9	72,424.759	0.12	10	72,424.759	0.14	1.000 (0.397–2.519)	>0.99
Tics/Tourette’s syndrome	132	71,554.879	1.84	174	71,214.176	2.44	1.375 (1.087–1.740)	0.008
Suicide	3	72,461.004	0.04	2	72,468.253	0.03	1.000 (0.141–7.099)	>0.99

PY: person-years; Rate: incidence rate (per 1000 person-years); 95% CI: 95% confidence interval; ADHD: attention deficit hyperactivity disorder; N/A: not applicable. Both groups were matched by propensity scores.

**Table 3:** Incidence and hazard ratios of neuropsychiatric disorder between non-montelukast and montelukast cohorts stratified by age and dosages of asthma medication, after propensity score matching.

	Asthmatic Children					
Variables	N	Event	PY	Rate	Crude Hazard Ratio (95% CI)	Adjusted Hazard Ratio (95% CI)
0–6 years						
Non-montelukast	3079	462	34,104.255	13.55	Reference	Reference
Low exposure (<101 days)	1902	195	17,814.132	10.95	0.810 (0.685–0.958) *	0.813 (0.688–0.961) *
High exposure (≥101 days)	1907	243	17,521.516	13.87	1.040 (0.891–1.215)	1.024 (0.877–1.197)
6–15 years						
Non-montelukast	3333	303	31,370.196	9.66	Reference	Reference
Low exposure (<63 days)	1627	157	15,285.665	10.27	1.054 (0.869–1.278)	1.049 (0.865–1.272)
High exposure (≥63 days)	1628	162	15,296.688	10.59	1.062 (0.877–1.285)	1.053 (0.870–1.274)
15–18 years						
Non-montelukast	207	34	1826.568	18.61	Reference	Reference
Low exposure (<56 days)	92	14	816.776	17.14	0.910 (0.488–1.696)	0.904 (0.484–1.691)
High exposure (≥56 days)	93	17	795.987	21.36	1.096 (0.612–1.962)	1.091 (0.608–1.957)

PY: person-years; Rate: incidence rate (per 1000 person-years); 95% CI: 95% confidence interval; * *p* < 0.05. Asthma control status was used for adjustment.

**Table 4:** Incidence and hazard ratios of Tics/Tourette’s syndrome between non-montelukast and montelukast cohorts stratified by age and dosages of asthma medication, after propensity score matching.

	Asthmatic Children					
Variables	N	Event	PY	Rate	Crude Hazard Ratio (95% CI)	Adjusted Hazard Ratio (95% CI)
0–6 years						
Non-montelukast	3079	90	36,481.724	2.47	Reference	Reference
Low exposure (<101 days)	1902	45	18,694.758	2.41	0.962 (0.673–1.376)	0.968 (0.677–1.385)
High exposure (≥101 days)	1907	61	18,663.809	3.27	1.328 (0.960–1.839)	1.288 (0.930–1.784)
6–15 years						
Non-montelukast	3333	42	32,993.367	1.27	Reference	Reference
Low exposure (<63 days)	1627	27	16,047.101	1.68	1.321 (0.815–2.143)	1.319 (0.813–2.139)
High exposure (≥63 days)	1628	41	15,941.376	2.57	2.006 (1.305–3.085) **	1.998 (1.299–3.074) **
15–18 years						
Non-montelukast	207	0	N/A	N/A	N/A	N/A
Low exposure (<56 days)	92	0	N/A	N/A	N/A	N/A
High exposure (≥56 days)	93	0	N/A	N/A	N/A	N/A

PY: person-years; Rate: incidence rate (per 1000 person-years); 95% CI, 95% confidence interval; ** *p* < 0.01. Adjusted for control. N/A: not applicable.

## Data Availability

The data presented in this study are available on request from the corresponding author. The data are not publicly available due to privacy and ethical restrictions.

## Supplementary Materials

The following supporting information can be downloaded at https://www.mdpi.com/article/10.3390/ph18030379/s1: Table S0: ICD codes of diagnosis for outcome and comorbidity variables. Table S1: Demographics and comorbidity differences between patients receiving montelukast integration treatments (Montelukast cohort) and those who are not (non-montelukast cohort) in children with asthma before propensity score matching. Table S2: Montelukast exposure days stratified by age. Table S3: Asthma control status and the association between montelukast use and neuropsychiatric disorders, including Tics/Tourette’s syndrome. Table S4: Incidence and hazard ratios of neuropsychiatric disorder between non-montelukast and montelukast cohorts stratified by gender, age, and dosages of asthma medication, after propensity score matching. Table S5: Incidence and hazard ratios of Tics/Tourette’s syndrome between non-montelukast and montelukast cohorts stratified by gender, age, and dosages of asthma medication, after propensity score matching. Table S6: The impact of corticosteroid dosage and duration on the development of Tics/Tourette’s syndrome among Montelukast users.

## Abbreviations

The following abbreviations are used in this manuscript:

DALYs	Disability-adjusted life years
LTMA	Leukotriene-modifying agent
LTRA	Leukotriene receptor antagonist
NHIRD	National Health Insurance Research Database
CysLT1R	Cysteinyl leukotriene receptor type 1
