# Supplementary material for: The Impact of Montelukast Duration on the Risk of Neuropsychiatric Disorders in Children with Asthma: A Population-Based Cohort Study
Source: Pharmaceuticals (Basel). 2025 Mar 7;18(3):379. doi: 10.3390/ph18030379 (PMC11946223; doi:10.3390/ph18030379)
Supplement: Supplementary file 1 [file pharmaceuticals-18-00379-s001.zip › pharmaceuticals-3483422-supplementary.pdf]

Table S0. ICD codes of diagnosis for outcome and comorbidity variables

Outcome variables	
Diagnosis	ICD 9 CM code
Attention deficit and hyperactivity disorder (ADHD)	314.0-314.9
Affective disorder	296.xx
Anxiety disorder	300.xx
Autism, autistic spectrum disorder	299.0x
Conduct disorder	312
Delay	315.0~315.9
Emotional Disturbance (childhood and adolescent)	313.80-313.89, 313.9
Personality disorder	301.xx
Psychosis	298.8, 298.9
Adjustment disorder	309.xx
Schizophrenia	295.0-295.9
Suicide	E950.x-E958.x
Tics/ Tourette's	307.2x
**Comorbidities variables**	
Diagnosis	ICD 9 CM code
Allergic rhinitis	477.9
Atopic dermatitis	691.8
Convulsions	780.39, 780.31
Epilepsy	345.x
Congenital hypothyroidism	243
Meningitis	320.xx, 321.xx, 322.xx
Juvenile idiopathic arthritis (JIA)	714.0,7142, 714.3x
Systemic lupus erythematous (SLE)	710.0
Dermatomyositis	710.3
Ankylosing spondylosis	720.0
Kawasaki disease	446.1
Child abuse	995.5x

Table S1. Demographics and comorbidity differences between patients receiving montelukast integration treatments (Montelukast cohort) or not (non-montelukast cohort) in children with asthma before propensity score matching.

	Asthma children				
	Non-montelukast cohort N= 8432		Montelukast cohort N= 14606		*P*
Variables	n	%	n	%	
Sex					
Girls	3444	40.8	6123	41.9	0.110
Boys	4988	59.2	8483	58.1	
Age, year, mean (SD) [#]	7.40 $\pm$ 3.97		5.25 $\pm$ 2.95		<0.001
< 6	3716	44.1	10332	70.7	<0.001
≥ 6, <15	4232	50.2	4080	27.9	
≥ 15	484	5.7	194	1.3	
Index year					
2004	2387	28.3	3345	22.9	<0.001
2005	2171	25.7	3731	25.5	
2006	1604	19.0	2845	19.5	
2007	1375	16.3	2642	18.1	
2008	895	10.6	2043	14.0	
Urbanization					
Urban	5102	60.5	7714	52.8	<0.001
Rural	2423	28.7	5305	36.3	
Offshore regions	907	10.8	1587	10.9	
Parents' Salary					
< 15840	8113	96.2	11457	99.0	<0.001
15841-25000	252	3.0	113	0.8	
> 25001	67	0.8	36	0.2	
Comorbidity					
Allergic rhinitis	6331	75.1	10175	69.7	<0.001
Atopic dermatitis	1534	18.2	3123	21.4	<0.001
Convulsion	36	0.4	110	0.8	0.003
Hypothyroidism	14	0.2	39	0.3	0.123
Epilepsy	92	1.1	145	1.0	0.476
Meningitis	48	0.6	55	0.4	0.035
JIA	5	0.1	3	<0.1	0.152
SLE	4	< 0.1	7	< 0.1	>0.999
Dermatomyositis	0	N/A	2	< 0.1	0.536
Spondylitis	0	N/A	0	N/A	N/A
Kawasaki	45	0.5	65	0.4	0.347
Abuse	2	< 0.1	0	N/A	0.134
Asthma medication used					
ICS	8409	99.7	5292	36.2	< 0.001
LABA	1383	16.4	1074	7.4	<0.001

SD: standard difference, *p* <0.05 means significant differences between two groups. JIA: juvenile idiopathic arthritis, SLE: systemic lupus erythematosus, ICS: inhaled corticosteroid, LABA: long-acting beta-2 agonist, N/A: not applicable

Table S2. Montelukast exposure days stratified by age

	Montelukast cohort	< 6 y/o	$\geq$ 6, <15 y/o	$\geq$ 15 y/o
Number of patients	7249	3809	3255	185
Average duration of use	154.21	183.97	122.6	97.46
25th percentile	28	40.50	28	28
Median	84	101	63	56
75th percentile	184	228	140	100
Mode	28	28	28	28
minimum	2	2	2	2
maximum	4308	4308	3024	1232

Table S3. Asthma control status and the association between montelukast use and neuropsychiatric disorders, Including Tics/Tourette’s syndrome.

Asthma children					
Variables	N	Event	PY	Rate	Crude Hazard Ratio (95% CI)
Neuropsychiatric disorders					
Well controlled					
Non-montelukast cohort	5012	493	46877.236	10.52	Reference
Montelukast cohort	4784	485	44749.536	10.84	1.029 (0.907-1.166)
Not well controlled					
Non-montelukast cohort	2237	306	20441.706	14.97	Reference
Montelukast cohort	2465	303	22828.365	13.27	0.887 (0.757-1.040)
Tics/Tourette's syndrome					
Well controlled					
Non-montelukast cohort	5012	77	49563.668	1.55	Reference
Montelukast cohort	4784	106	47026.72	2.25	1.445 (1.077-1.937)*
Not well controlled					
Non-montelukast cohort	2237	55	21991.947	2.50	Reference
Montelukast cohort	2465	68	24179.185	2.81	1.110 (0.778-1.584)

PY: person-years; Rate, incidence rate (per 1000 person-years); 95% CI, 95% confidence interval; *$p < 0.05$

Table S4. Incidence and hazard ratio of neuropsychiatric disorder between non-montelukast and montelukast cohorts stratified by gender, age and dosages of asthma medication, after propensity score matching.

Asthma children						
Variables	N	Event	PY	Rate	Crude Hazard Ratio (95% CI)	Adjusted Hazard Ratio (95% CI)
Boys						
0-6 years						
Non-Montelukast	2178	351	18,711.198	18.76	reference	reference
Low exposure (< 101 days)	1082	130	10,010.664	12.99	0.724 (0.592-0.886)*	0.726 (0.594-0.888)**
High exposure (≥ 101 days)	1158	191	10,372.206	18.41	1.038 (0.870-1.238)	1.027 (0.861-1.226)
6-15 years						
Non-Montelukast	1985	192	18,559.75	10.34	reference	Reference
Low exposure (< 63 days)	965	94	9042.05	10.40	0.990 (0.774-1.267)	0.986 (0.770-1.261)
High exposure (≥ 63 days)	972	99	9127.08	10.85	1.106 (0.797-1.295)	1.010 (0.792-1.280)
15-18 years						
Non-Montelukast	108	11	984.744	11.17	reference	reference
Low exposure (< 56 days)	47	6	423.141	14.18	1.221 (0.451-3.307)	1.140 (0.416-3.120)
High exposure (≥ 56 days)	54	6	476.766	12.58	1.081 (0.399-2.926)	1.021 (0.374-2.785)
Girls						
0-6 years						
Non-Montelukast	1531	111	14,471.012	7.67	reference	reference
Low exposure (< 101 days)	820	65	7802.3	8.33	1.093 (0.805-1.485)	1.097 (0.807-1.489)
High exposure (≥ 101 days)	749	52	7150.703	7.27	0.974 (0.700-1.354)	0.957 (0.688-1.333)
6-15 years						
Non-Montelukast	1348	111	12,810.044	8.67	reference	reference
Low exposure (< 63 days)	662	63	6241.336	10.09	1.162 (0.853-1.582)	1.156 (0.849-1.575)
High exposure (≥ 63 days)	656	63	6169.024	10.21	1.136 (0.833-1.547)	1.121 (0.823-1.528)
15-18 years						
Non-Montelukast	99	23	830.412	27.70	reference	reference
Low exposure (< 56 days)	45	8	390.915	20.46	0.741 (0.331-1.657)	0.744 (0.332-1.666)
High exposure (≥ 56 days)	39	11	312.039	35.25	1.201 (0.586-2.465)	1.202 (0.586-2.466)

PY: person-years; Rate, incidence rate (per 1000 person-years); 95% CI, 95% confidence interval; *$p < 0.05$, **$p < 0.01$. Asthma control status was used for adjustment.

Table S5. Incidence and hazard ratio of Tics/ Tourette's disorder between non-montelukast and montelukast cohorts stratified by gender, age and dosages of asthma medication, after propensity score matching.

Asthma children						
Variables	N	Event	PY	Rate	Crude Hazard Ratio (95% CI)	Adjusted Hazard Ratio (95% CI)
Boys						
0-6 years						
Non-Montelukast	2178	70	21,307.374	3.29	reference	reference
Low exposure (< 101 days)	1082	38	10,549.5	3.60	1.068 (0.719-1.585)	1.073 (0.723-1.593)
High exposure (≥ 101 days)	1158	51	11,236.074	4.54	1.381 (0.963-1.981)	1.348 (0.939-1.395)
6-15 years						
Non-Montelukast	1985	33	19,587.98	1.68	reference	reference
Low exposure (< 63 days)	965	21	9478.23	2.22	1.308 (0.757-2.261)	1.301 (0.753-2.249)
High exposure (≥ 63 days)	972	30	9471.168	3.17	1.858 (1.133-3.046)*	1.842 (1.123-3.022)*
Girls						
0-6 years						
Non-Montelukast	1531	30	15,170.679	1.98	reference	reference
Low exposure (< 101 days)	820	7	8143.42	0.86	0.652 (0.276-1.542)	0.654 (0.276-1.546)
High exposure (≥ 101 days)	749	10	7424.837	1.35	1.024 (0.479-2.188)	1.009 (0.471-2.165)
6-15 years						
Non-Montelukast	1348	9	13,403.164	0.67	reference	reference
Low exposure (< 63 days)	662	6	6564.392	0.91	1.370 (0.488-3.850)	1.375 (0.490-3.864)
High exposure (≥ 63 days)	656	11	6470.784	1.70	2.538 (1.052-6.126)*	2.567 (1.063-6.199)*

PY: person-years; Rate, incidence rate (per 1000 person-years); 95% CI, 95% confidence interval; *$p < 0.05$. Asthma control status was used for adjustment.

Table S6. The impact of corticosteroid dosage and duration on the development of Tics/Tourette's disorder among Montelukast users

	Tics/Tourette's syndrome	Number	Mean	t-test	*p* value
Steroid dosages (mg)	-	7075	37.38	0.33	0.74
	+	174	7.47		
Steroid durations (days)	-	7075	35.28	0.31	0.75
	+	174	30.82		
